# Medical College and Hospital Notes

**Published:** 1898-04

**Authors:** 


### Medical College and Hospital Notes.

The Medical Department of the University of Buffalo will hold its fifty-second annual commencement Tuesday, April 26, 1898. The alumni association will hold its twenty-fourth annual meeting at Alumni Hall in the college building during the morning and afternoon of the same day. The annual banquet of the faculty, officers and alumni will be given at the Ellicott Club in the evening. An elaborate program is in preparation, which will embrace a number of interesting papers and addresses. The alumni should attend these annual exercises in full force.

---

The Medical Department of Niagara University will hold its thirteenth annual convocation Wednesday, May 11, 1898. The alumni association will hold its annual meeting at the college building on Ellicott street in the morning and afternoon, and the annual banquet in the evening. A program is preparing that will prove full of interest, embracing papers, addresses and speeches. All alumni and others interested in these ceremonies are earnestly and cordially invited to attend.

---

THE New York Skin and Cancer Hospital opened its new building, corner of Second avenue and Nineteenth street, March 5, 1898. It is a modern fire-proof structure of four stories and basement, containing about sixty beds for patients, seven of which are in private rooms. On the top floor there is a modern operating room, well equipped, the Worden ward for children, and most of the private rooms. On the next two floors are four wards, for male and female patients with diseases of the skin and cancer. On the ground floor is the large dispensary room, with consulting rooms, drug room, office, reception room, and the like. In the basement there is a complete set of baths, including Turkish, Russian and plunge, besides the kitchen, laundry and dining room. The lighting, heating and ventilation are of the most approved kind.

The occupation of the new building marks the fifteenth anniversary of the institution, as the first patient was admitted to the old building in Thirty-fourth street, on February 26, 1883. Dr. A. Jacobi, president of the medical board of the hospital, made brief introductory remarks at the ceremony, and Dr. L. Duncan Bulkley, who was the originator of the hospital, and is now acting as its secretary, made an address on The care of diseases of the skin and cancer in New York and elsewhere. He called attention to the fact that the old city of New York had increased in size, in the last ten years, at the rate of 50,000 inhabitants yearly, and suggested that hospital accommodations during this time had not increased proportionately. In regard to special hospitals he mentioned four or five for diseases of the eye and ear, several for diseases of women, several lying-in hospitals, a number for infants and children, two for the crippled, one for the throat, and public and private hospitals for contagious diseases, but stated that diseases of the skin and cancer had been neglected, until the establishment of the New York skin and cancer hospital fifteen years ago.

Paris has the great Hôpital St. Louis, with 500 to 600 beds for skin diseases, Vienna 200 to 300 beds, also there are large special accommodations for this class of patients in Berlin, Prague, Budapest, Copenhagen, Stockholm, St. Petersburg, Moscow, Rome, Palermo, Padua and many other cities, and twelve special hospitals for skin diseases in Great Britain. Relatively little had been done for cancer, the London cancer hospital standing almost alone.

Dr. Bulkley gave an account of the formation of the hospital society, and of the early struggles of the institution, and paid a warm tribute to Mrs. D. T. Worden, the secretary of the Ladies' Auxiliary Board, who died in 1887, and for whom the children's ward in the new hospital has been named.

During the past fifteen years the hospital had received some $375,000 from all sources, of which $75,000 had come from the board of patients and sale of drugs in the dispensary. Of the $300,000 which had been donated from outside, the hospital now had $25,000 invested as endowments of five free beds, and had paid $150,000 for the land and building, which was practically free from debt. He then made a strong appeal for funds for permanent endowment and for current expenses, expressing a strong desire for many small contributions from those who should take a personal interest in the work.

During the past fifteen years the hospital has treated 25,031 cases, of which 3,010 were in the cancer department and 22,021 in the skin department. Most of these had been treated as out patients, 22,159 having come to the hospital for treatment 118,154 times. For them 132,263 prescriptions were written by the physicians and surgeons and filled by the hospital druggist. In the beds of the institution there had been treated 2,872 persons, who had spent the enormous total of 165,077 days in the hospital, and of these "hospital days" 117,658 were entirely free. Not only had there been patients of all possible creeds and nationalities, but also patients from almost every state and territory in the union, as this was the only institution of the kind in the United States. He, therefore, appealed strongly for contributions from outside of New York city.

In conclusion Dr. Bulkley called attention to the educational advantages of the hospital, and referred to the thirty young men who had served on its house staff during the past fifteen years, whereby they had received an education in these diseases not to be obtained elsewhere in this country, and second only to prolonged study abroad. He also stated that it was proposed by the management to have clinical lectures in the hospital, free to physicians, where the diagnosis and treatment of the diseases cared for in the hospital could be learned. To aid in this the hospital had the loan of a large collection of wax models from Paris, and several thousand plates and pictures, to be used in the lectures. He remarked that skin diseases were often thought to be unimportant, but felt sure that no one witnessing the work done in the hospital would longer entertain such an opinion. Many of the cases had been horrible in the extreme, causing years of intense suffering and sometimes ending in distressing death.

---

THE Jennie Casseday Free Infirmary for Women, on Sixth street, opposite St. James's Court, has been sold by the King's Daughters to Dr. Lewis S. McMurtry. The infirmary was founded, owned and controlled by the King's Daughters, of Louisville, Ky. It was formally opened for the reception of patients April 12, 1892. The object of the infirmary was the treatment of diseases peculiar to women. All the rooms were furnished by individuals or circles of King's Daughters. The house was equipped throughout in accordance with the most approved methods of hospital management.

Shortly after the infirmary was opened, Dr. L. S. McMurtry was elected surgeon in charge by the lady board of managers. Under his direction the institution gained a wide reputation in all parts of the country. From the day the infirmary was opened it has been filled with patients. The income from the private department has always supported the institution, but there had been a debt on the property which the managers found it embarrassing to meet; therefore, the property, including the equipments, was offered for sale and Dr. McMurtry became the purchaser. The institution will be conducted just as before and will bear its original name.

---

THE Manhattan Eye and Ear Hospital in its twenty-eighth annual report states that the work of the hospital has increased to such an extent that it was found necessary, not infrequently, to perform operations upon the eyes of one patient, and upon the ears, throat or nose of another, at the same time and in the same operating room. In consequence of the inconvenience and embarrassment thus occasioned, the directors decided to do away with the department for diseases of the nervous system, and to convert the large room formerly occupied by that department into an operating-room for the departments of the ear, throat and nose. During the year the capacity of the hospital was further increased by the utilisation of an adjoining building. In the report attention is particularly called to the care that is exercised in excluding unworthy patients who are able to pay ; it being stated that 10 per cent. were excluded by the superintendent during the year, who acknowledged that they were able to pay for medical or surgical treatment.

